# Influence of Placental Abnormalities and Pregnancy-Induced Hypertension in Prematurity Associated with Various Assisted Reproductive Technology Techniques

**DOI:** 10.3390/jcm10081681

**Published:** 2021-04-14

**Authors:** Judy E. Stern, Chia-ling Liu, Sunah S. Hwang, Dmitry Dukhovny, Leslie V. Farland, Hafsatou Diop, Charles C. Coddington, Howard Cabral

**Affiliations:** 1 Department of Obstetrics & Gynecology, Dartmouth-Hitchcock, Lebanon, NH 03756, USA; 2 Bureau of Family Health and Nutrition, Massachusetts Department of Public Health, Boston, MA 02108, USA; liu_chialing@yahoo.com; 3 Section of Neonatology, Department of Pediatrics, University of Colorado School of Medicine, Aurora, CO 80045, USA; Sunah.hwang@childrenscolorado.org; 4 Division of Neonatology, School of Medicine, Oregon Health & Science University, Portland, OR 97239, USA; dukhovny@ohsu.edu; 5 Epidemiology and Biostatistics Department, Mel and Enid Zuckerman College of Public Health, University of Arizona, Tucson, AZ 85724, USA; lfarland@email.arizona.edu; 6 Division of Maternal and Child Health Research and Analysis, Bureau of Family Health and Nutrition Massachusetts Department of Public Health, Boston, MA 02108, USA; Hafsatou.Diop@state.ma.us; 7 Department of Obstetrics and Gynecology, University of North Carolina, Charlotte, NC 28204, USA; Charles.Coddington@atriumhealth.org; 8 Department of Biostatistics, Boston University School of Public Health, Boston, MA 02118, USA; hjcab@bu.edu

**Keywords:** assisted reproductive technology, in vitro fertilization, mediation, placental abnormalities, pregnancy-induced hypertension, prematurity

## Abstract

Objective. Assisted reproductive technology (ART)-treated women exhibit increased risk of premature delivery compared to fertile women. We evaluated whether ART treatment modalities increase prematurity and whether placental abnormalities and pregnancy-induced hypertensive (PIH) disorders mediate these risks. Method(s): This retrospective study of ART-treated and fertile deliveries (2004–2017) used an ART-cycle database linked to Massachusetts birth certificates and hospital discharges. Outcomes of late preterm birth (LPTB: 34–36 weeks gestation) and early preterm birth (EPTB: <34 weeks gestation) were compared with term deliveries (≥37 weeks gestation) in ART-treated (linked to the ART database) and fertile (no indicators of infertility or ART) deliveries. ART treatments with autologous oocyte, donor oocyte, fresh or frozen embryo transfer (FET), intracytoplasmic sperm injection (ICSI) and no-ICSI were separately compared to the fertile group. Adjusted odds ratios (AOR) were calculated with multivariable logistic regression: placental abnormalities or PIH were quantified in the pathway as mediators. Results: There were 218,320 deliveries: 204,438 fertile and 13,882 ART-treated. All treatment types increased prematurity (AOR 1.31–1.58, LPTB; AOR 1.34–1.48, EPTB). Placental abnormalities mediated in approximately 22% and 38% of the association with LPTB and EPTB, respectively. PIH mediated 25% and 33% of the association with LPTB and EPTB in FET and donor oocyte cycles, more than other treatments (<10% LPTB and <13% EPTB). Conclusions: ART-treatment and all ART modalities increased LPTB and EPTB when compared with fertile deliveries. Placental abnormalities modestly mediated associations approximately equally, while PIH was a stronger mediator in FET and donor oocyte cycles. Reasons for differences require exploration.

## 1. Introduction

Assisted reproductive technology (ART treatment), defined as treatments including manipulation of oocytes and/or embryos in vitro [1], is associated with an increase in adverse pregnancy and delivery outcomes. Some of these adverse outcomes are a direct result of the increased rate of multiple gestation in these pregnancies [2,3]; however, it is now well established that rates of adverse outcomes are elevated even in singleton deliveries [4,5]. Adverse outcomes that have been previously studied include pregnancy-associated abnormalities of gestational diabetes, pregnancy-associated hypertension/preeclampsia/eclampsia, placental abnormalities, as well as delivery outcomes of low birthweight, prematurity, and perinatal mortality [6,7,8].

Although there has been extensive study of adverse pregnancy outcomes associated with ART, there remains a question about why these adverse outcomes occur. One hypothesis is that underlying infertility rather than ART is associated with the adverse outcomes and it is clear from prior studies that at least a portion of the risk is associated with these underlying factors [9,10]. Nevertheless, even when compared to patients with prior infertility or other fertility treatments, many of these adverse outcomes persist among women undergoing ART. Recently, we chose to look at the outcome of prematurity and to evaluate various factors that might influence prematurity following ART treatment and subfertility [11]. We demonstrated that placental abnormalities mediate a proportion of the effect on prematurity in both ART and subfertile deliveries but that the extent to which this occurs is more pronounced following ART. A mediator is a factor (in this case placental problems) on the pathway between the exposure (in this case ART) and the outcome (in this case prematurity) [12]. The next question to ask is whether mediation of prematurity by placental abnormalities is equally influenced by different ART treatment modalities. Prior research has also suggested an increased risk for pregnancy-induced hypertensive (PIH) disorders for some ART-treated women. This occurs in pregnancies conceived via frozen embryo transfer (with both autologous or donor oocyte) and fresh donor oocyte embryo transfer [13]. Our prior study on prematurity had also found that, in addition to placental problems, pregnancy-induced hypertension (PIH) had the largest effect on prematurity in adjusted logistic regressions [12]. We thus believed a study of mediation by PIH, as well as placental problems, to be warranted.

In this study, we used a larger dataset than that of our original mediation study, to evaluate individual ART treatment procedures including the use of autologous oocytes, donor oocytes, fresh and frozen embryo transfer (FET), and the use of intracytoplasmic sperm injection (ICSI) or standard insemination. We evaluated whether these procedures exhibited differences in the extent to which placental abnormalities and PIH mediated the risk of prematurity.

## 2. Materials and Methods

This was a retrospective cohort study that used data from (1) the Society for Assisted Reproductive Technology Clinic Outcome Reporting System (SART CORS), (2) the Massachusetts-based Pregnancy to Early Life Longitudinal (PELL) data system, and (3) the All Payers Claims Database (APCD). The study was conducted under Memoranda of Understanding among SART, the Massachusetts Department of Public Health (MDPH), the Center for Health Information and Analysis (CHIA) and the project principal investigators. Institutional Review Board approval was obtained from MDPH (249896 first approved 15 July 2010:268998 first approved 19 March 2013:257261 first approved 25 March 2015) and Dartmouth-Hitchcock Health (CR00005901 with initial approval by the Dartmouth College Committee for the Protection of Human Subjects as study 23205 on 10 February 2012).

### 2.1. Data Sources

SART CORS contains cycle-based ART data from close to 90% of ART clinics in the US and all clinics in Massachusetts. It contains demographics, infertility diagnoses, ART treatment, pregnancy, and outcome data for individual ART cycles. Data entered into SART CORS by the clinics are reported to the Centers for Disease Control and Prevention in compliance with the Fertility Clinic Success Rate and Certification Act of 1992 (Public Law 102-493). SART CORS data are validated annually through on-site visits that review a randomly selected group of clinics in which reported data are compared with information in patients’ charts. In 2017, most data fields selected for validation were found to have discrepancy rates of ≤ 5% [14].

PELL is a Massachusetts population-based data system containing data from birth certificates, fetal death certificates and corresponding delivery hospital discharge records as well as ongoing hospital utilization records (hospital admissions, observational stays, and emergency room visits) for mothers and infants over time. Data have been linked for 98% of births and fetal deaths for individual women and their children since 1998. PELL data are linked through randomly generated unique IDs for mothers and infants. The Massachusetts Department of Public Health (MDPH) and CHIA are the custodians of the PELL data which are housed at MDPH.

The APCD is a comprehensive claims database that houses insurance claims from public and private insurance payers providing insurance to Massachusetts residents and employees. The database includes medical insurance claims including those for outpatient infertility treatment and was used to exclude additional infertile women from the fertile group if delivery occurred between 1 January 2013 and 31 December 2017. We have previously published on some of the strengths and limitations on use of this database for fertility research [15].

We linked data from SART CORS and PELL to create the Massachusetts Outcome Study of Assisted Reproductive Technology (MOSART) for Massachusetts resident women delivering in Massachusetts hospitals from 1 January 2004 through 31 December 2017. The linkage algorithm includes mother’s first name and last name, and date of birth; father/partner’s last name, baby’s date of birth, plurality, and infant sex, as previously reported [16]. The 2004–2017 linkage rate data were 91.5% overall and 94.9% for deliveries in which both mother’s zip code and clinic were located in Massachusetts. The MOSART database from 2013 through 2017 was further linked to the APCD. The linkage was performed by CHIA using mother’s date of birth, last and first names, and zip codes. Overall, 98.7% of the MOSART mothers were linked to APCD.

### 2.2. Study Sample

Our study sample included first, singleton, live birth deliveries to women from MOSART who had private insurance and were ≥18 years of age. We excluded gestational carriers, multiparous women, and women who had stillbirths. Women were classified as ART-treated if the delivery was linked to the SART CORS database. They were considered fertile if they were not linked to SART CORS and did not have any of our previously defined parameters of subfertility [17], nor did they have an outpatient diagnosis of infertility (by International Classification of Diseases (ICD) 9 codes 628 and V230; ICD 10 O09.00-O09.03 and N97.0–N97.9) in APCD. ART treatment groups of fresh or frozen embryo, autologous or donor egg, and ICSI (some or all) or no ICSI, were defined from cycle-specific treatment parameters within SART CORS. These categories are not mutually exclusive (i.e., autologous oocyte cycles can be fresh or frozen and can use ICSI or not use it, and so forth).

### 2.3. Outcomes and Covariates

Our outcome variable of prematurity was calculated from gestational age obtained from the birth certificates and determined from clinical dating ultrasound modified by estimated last menstrual period where needed. We included only those gestational ages in the range of 17–44 weeks. Outcomes were classified as term deliveries (≥37 weeks gestation), late preterm deliveries (LPTB: 34–36 weeks gestation), and early preterm deliveries (EPTB: <34 weeks gestation). We obtained the following additional covariates from birth certificates: maternal and paternal age, race/ethnicity and education, country of origin, prior gravidity, year of delivery, and infant sex. Other covariates were obtained from hospital discharges or a combination of birth certificate and hospital discharge information. Prior uterine surgery was determined on the basis of data from hospital inpatient, observation, and emergency records prior to conception. A combination of birth certificates, fetal death certificates, and hospital delivery discharge records were used to define chronic hypertension and diabetes, placental problems (abruptio placentae, placenta previa, vasa previa, and placenta accreta), premature rupture of membranes, and method of delivery. Pregnancy risk including gestational diabetes, PIH (pregnancy hypertension/preeclampsia/eclampsia), and pregnancy-associated bleeding were determined from birth and fetal death certificates and hospital inpatient, observation, and emergency records 280 days prior to delivery (See Supplemental Table S1 for ICD 9 and 10 codes).

### 2.4. Statistics

Adjusted odds ratios (AOR) and 95% confidence intervals (95% CI) for each outcome comparing the LPTB and EPTB deliveries to term deliveries were estimated using logistic regressions, adjusting for mother’s age, race/ethnicity, education, chronic diabetes and hypertension, prior uterine surgery, gravidity, gestational diabetes, pregnancy hypertension, placental problems, pregnancy-associated bleeding, father’s age, race and education, and infant sex. Each ART treatment group was compared with the fertile group. All analyses were performed in SAS software 9.4 (SAS Institute, Cary, NC, USA), and the PROC CAUSALMED procedure was used for mediation analyses to quantify direct and indirect effects of ART treatments on LPTBs and EPTBs. To estimate the natural direct and indirect effects in mediation analysis, we assumed no unmeasured confounding between the exposure and outcome relationship, between the exposure and mediator relationship, and between the mediator–outcome relationship and no mediator–outcome confounder that is affected by the exposure [12]. However, if the results of an analysis indicated that the percentage of effects due to interaction was statistically significant, we altered the model to include interaction effects [18].

## 3. Results

Our study sample included 218,320 deliveries. Among all deliveries, 5277 (2.42%) had placental problems, of which 16.16% were LPTB and 11.64% were EPTB, and 25,172 (11.53%) had PIH, of which 9.81% were LPTB and 4.27% were EPTB. With regard to fertility groups, there were 204,438 (93.64%) in the fertile group, of which 4.71% had LPTB and 1.48% EPTB, while the ART group included 13,882 (6.36%) deliveries, of which 7.50% were LPTB and 2.98% EPTB (Table 1). In the fertile group, 2.13% had placental problems and 11.31% had PIH, whereas in the ART group 6.66% had placental problems and 14.80% had PIH. The different treatment groups within the ART group had somewhat differing prematurity rates, but AORs were similar for all (Table 2).

The mediation analysis with placental problems as mediator is shown in Table 3. Approximately 37% of the overall association between any ART procedures and EPTB and 23% of the association between any ART procedures and LPTB can be attributed to placental problems. As can be seen, different ART procedures had similar patterns for direct and indirect effects. Of all the IVF modalities, placental problems mediated the largest proportion of the association between ICSI and EPTB (44%).

Table 4 presents the mediation analysis with PIH as mediator, demonstrating that treatment groups showed differences in percent mediation according to treatment types. The highest percentage mediation was found for frozen embryo transfer (25.9% LPTB; 32.6% EPTB) and donor egg cycles (24.5% LPTB; 35% EPTB).

Analyses showed significant interaction, but the estimates of the mediation effects, their 95% confidence intervals, and *p*-values were essentially unchanged when comparing models with and without the interaction included (data available from the authors on request).

## 4. Discussion

In this study, we found that prematurity was increased in ART pregnancies when compared with pregnancies to fertile women without fertility treatment. The finding of elevated rates of prematurity in ART is consistent with prior results [5,13,19,20]. We further demonstrated that the effect of ART on prematurity was partially mediated by both placental abnormalities (including: abruptio placentae, placenta previa, vasa previa, and placenta accreta) and PIH. ART pregnancies from fresh and frozen embryo transfer cycles, use of autologous or donor egg, and ICSI or no ICSI all showed comparable percent mediation by placental abnormalities. By contrast, the influence of PIH on the association between ART and prematurity varied across ART modalities. The percentage mediated was greater for frozen embryo transfer and use of donor eggs than for the other treatment types.

The cause of prematurity, which affects approximately 10% of US births [21], is still incompletely understood [22]. The risk factors for preterm birth are multifactorial and include a range of biological and environmental factors such as maternal age, race/racism, education, body mass index, a history of smoking, low socioeconomic status, and periodontal disease [23] as well as psychological stress [24]. Maternal health prior to and during the pregnancy, including conditions such as hypertension and diabetes, in addition to other maternal diseases are also known risk factors for preterm birth. Among the triggers for prematurity are placental abnormalities and pregnancy-induced hypertension including preeclampsia-eclampsia [22,23]. Risks for prematurity increase in ART pregnancies partially because some of the aforementioned risk factors (age, chronic diabetes, chronic hypertension) are increased in these women [25]. Further, the risks for prematurity increase exponentially in multiple as compared with singleton pregnancies, and it is well known that ART treatment increases the risk for multiple pregnancies [3]. That is why only singleton deliveries were used in this study.

It has long been established that a variety of adverse obstetric outcomes are increased in singleton ART deliveries as compared with those to both fertile women and women with infertility or non-ART infertility treatment [4,5,6,8,19,26,27]. Adverse outcomes reported to be elevated have included PIH, gestational diabetes, and placental abnormalities including placental previa and vasa previa [13,28,29]. Delivery outcomes reported to be elevated have included low birthweight, prematurity, small for gestational age and, following frozen embryo transfer, large-for-gestational-age babies [4,5,6,8,13,30,31,32,33,34]. Differences are found when the comparison group consists of fertile women, but also when infertile/subfertile women and women treated for infertility with non-ART treatments are compared [8,10,13,20]. For example, Luke et al. demonstrated an increase in placental abnormalities and PIH in singletons in both ART-treated (aOR 2.81, 95% CI 2.57–3.08 for placental; aOR 1.22, 95% CI 1.15–1.28 for PIH) and subfertile (aOR 1.44, 95% CI 1.26–1.66 for placental; aOR 1.12, 95% CI 1.05–1.20 for PIH) women [13]. This same study also showed these women to have increases in gestational diabetes and bleeding during pregnancy. Since these increases are seen in subfertile as well as ART-treated women, it is likely that adverse outcomes arise, at least in part, from underlying health conditions in women who undergo ART [9,10]. Nevertheless, women who utilize ART treatment consistently demonstrate rates for these conditions that are somewhat elevated over those of infertile women. Whether this reflects an effect of the use of ART treatment, or the fact that ART is often used for women with more severe infertility and subsequent abnormal underlying conditions, is not known.

Different ART treatment modalities have been shown to differ in rates of adverse pregnancy outcomes. Luke et al. [7] demonstrated that although multiple birth is the greatest risk factor for adverse outcomes in ART pregnancies, there is an increased risk of PIH and preterm birth following the use of donor oocytes as well as a higher rate of small for gestational age deliveries following ICSI. Several meta-analyses have demonstrated that frozen embryo transfer has a lower risk of prematurity and low birthweight than fresh embryo transfer [6,35]. Luke et al. [7] showed that, among ART deliveries, there is a higher rate of PIH in frozen embryo transfer versus fresh (aOR 1.30, 95% CI 1.08–1.57) and donor oocyte versus fresh (aOR 1.87, 95% CI 1.45–2.42). Similarly, Barsky et al. showed preeclampsia to be elevated threefold in pregnancies following frozen embryo transfer using vitrified embryos, as compared with fresh embryo transfer [36].

We have previously demonstrated mediation of prematurity by placental problems in ART deliveries as compared with fertile and subfertile deliveries [11]. The percent mediation by placental problems in ART deliveries was 15% for LPTB and 32% for EPTB; however, among subfertile women with fertility treatment, placental problems only contributed to 7% of the association with LPTB and 12% for EPTB. Similarly, for subfertile women without fertility treatment, placental problems only accounted for 7% of the association for LPTB and 14% for EPTB. These findings suggest that the ART procedure itself may contribute to prematurity through placental abnormalities. Our results in the current study are consistent with this observation in that all ART treatment types studied showed similar magnitude of mediation by placental abnormalities. Whether some aspect of the ART procedure itself contributes to placental abnormalities leading to prematurity remains to be investigated. Such aspects could potentially involve the laboratory aspects of in vitro culture itself or abnormalities introduced during the transfer of the embryos to the uterus.

By contrast, mediation by PIH differed by treatment type. In this study, both frozen embryo transfer and donor oocyte deliveries showed a higher percentage mediation by PIH on the risk of prematurity than the other modalities studied. Previous studies have suggested that frozen embryo transfer results in higher rates of pregnancy-induced hypertensive disorders including preeclampsia than does fresh embryo transfer [37]. This is true for our data set as well with 13.21% of fresh embryo transfer and 20.13% of frozen embryo transfer being PIH deliveries (crude OR 1.66, 95% CI 1.49–1.84). As hypothesized in the literature, one reason for this increase could be the absence of the corpus luteum in frozen embryo transfer cycles in which the endometrium is generally prepared using estrogen and progesterone, which bypasses the formation of the corpus luteum [38,39]. Under this hypothesis, other factors such as the vasoactive products relaxin, vascular endothelial growth factor, and angiogenic metabolites of estrogen, produced by the corpus luteum, are absent from stimulated cycles and this absence might lead to the increase in PIH. Notably, the absence of the corpus luteum is also a factor in donor oocyte cycles where the uterine endometrium of the recipient of the embryo made from the donor oocyte is prepared for receipt of the embryo using the same estrogen and progesterone protocols that are used for frozen embryo cycles [40,41]. These findings suggest that these protocols used in frozen embryo transfer and donor oocyte cycles could increase prematurity through association with PIH. Our analyses showed that the results of the mediation analysis remained significant even when interaction models were run with PIH as mediator. Nevertheless, we hypothesize that the influence of this mediator could be complex as there could also be effects of the treatments on PIH itself as well as unmeasured or unknown confounding factors that could not be taken into account in this study. Complex interactions were not planned within the scope of this paper using the available data and will need to be evaluated as elements of focus in future studies.

Our study had strengths and limitations. The strengths include the large sample size that allowed us to evaluate mediation, not only in grouped ART deliveries but also in several ART treatment types separately as these compare with the fertile population. Our linked database also provided us with extensive information on maternal conditions from both the birth certificates and hospital discharges. Further, we have the strength of having detailed cycle-based information from the SART CORS. Limitations include the retrospective design of the study resulting in some important covariates such as smoking, diet, and BMI being unavailable or inaccurate and thus not available for analysis. Thus, while many known confounders were adjusted for in these analyses, there are unmeasured or unknown confounding factors that were not taken into account. It is also possible that the fertile group contained some deliveries to women with infertility, however, these would serve only to attenuate the observed differences. We identified covariates from the birth certificates and hospitalization resulting in the possibility that we missed women with conditions diagnosed and treated solely on an outpatient basis. APCD outpatient claims were not used to obtain these variables because their accuracy is unknown. Finally, the study was performed using records of Massachusetts deliveries only, and thus, results may not be generalizable to all states and countries.

## 5. Conclusions

In summary, we found that ART increased both LPTB and EPTB when compared with deliveries to fertile women, and that this increase was mediated by both placental abnormalities and PIH. Placental abnormalities mediated fresh and frozen embryo transfer, autologous and donor oocyte cycles, and ICSI and non-ICSI cycles approximately equally, while PIH was a stronger mediator of frozen embryo transfer and donor oocyte cycles than of other treatment types. This difference might relate to the use of estrogen- and progesterone-stimulated cycles in these cases. Further study will be needed to determine the causal mechanisms of how ART disrupts placentation and blood pressure so that interventions that decrease placental abnormalities can be developed to reduce the risk for prematurity among women who undergo ART.

## Figures and Tables

**Table 1:** Term, late preterm and early preterm delivery in deliveries with placental problems and PIH and in fertile, ART-treated and ART subgroups.

Condition or Treatment	Total		Term		LPTB		EPTB	
	N	%	N	%	N	%	N	%
**Total**	218,320	100.00	204,207	93.54	10,662	4.88	3451	1.58
Pregnancy-Induced Hypertension (PIH)	25,172	100.00	21,627	85.92	2469	9.81	1076	4.27
Abruptio placentae	2511	100.00	1602	63.80	433	17.24	476	18.96
Placenta previa	1677	100.00	1247	74.36	320	19.08	110	6.56
Vasa Previa	117	100.00	49	41.88	57	48.72	11	9.40
Placenta accreta	1128	100.00	982	87.06	93	8.24	53	4.70
Placenta Problems (Composite of abruptio placentae, placental previa, vasa previa, placenta accreta)	5277	100.00	3810	72.20	853	16.16	614	11.64
Fertility Group								
Fertile	204,438	100.00	191,779	93.81	9621	4.71	3038	1.49
ART	13,882	100.00	12,428	89.53	1041	7.50	413	2.98
Fresh	10,787	100.00	9685	89.78	789	7.31	313	2.90
Frozen	2941	100.00	2612	88.81	235	7.99	94	3.20
Autologous egg	12,633	100.00	11,353	89.87	918	7.27	362	2.87
Donor egg	1248	100.00	1074	86.06	123	9.86	51	4.09
ICSI ^2^	5050	100.00	4527	89.64	378	7.49	145	2.87
No ICSI ^1^	5887	100.00	5285	89.77	428	7.27	174	2.96

^1^ LPTB = Late preterm birth; EPTB = Early preterm birth; ^2^ Includes fresh and combo fresh + frozen cycles.

**Table 2:** Crude and adjusted odds ratios for ART and ART modalities.

Condition or Treatment	Crude Odds Ratios				Adjusted Odds Ratios			
	LPTB		EPTB		LPTB		EPTB	
	OR	95% CI	OR	95% CI	AOR ^1^	95% CI	AOR ^1^	95% CI
Pregnancy Complications								
PIH	2.54	2.43–2.67	3.83	3.55–4.12	2.37	2.26–2.49	3.42	3.17–3.70
Placental abnormalities	4.57	4.24–4.94	11.38	10.37–12.49	4.35	4.02–4.70	10.26	9.31–11.32
Fertility Group								
Fertile	ref		ref		ref		ref	
ART								
All ART	1.67	1.56–1.78	2.10	1.89–2.33	1.36	1.27–1.47	1.45	1.29–1.64
Fresh	1.62	1.51–1.75	2.04	1.81–2.30	1.38	1.27–1.49	1.44	1.26–1.64
Frozen	1.79	1.57–2.05	2.27	1.85–2.80	1.31	1.14–1.51	1.39	1.11–1.74
Autologous egg	1.61	1.50–1.73	2.01	1.80–2.25	1.34	1.24–1.44	1.42	1.26–1.61
Donor egg	2.28	1.89–2.75	3.00	2.26–3.98	1.58	1.27–1.97	1.48	1.05–2.08
ICSI	1.66	1.50–1.85	2.02	1.71–2.40	1.39	1.24–1.56	1.34	1.11–1.61
No ICSI	1.61	1.46–1.79	2.08	1.78–2.43	1.35	1.21–1.50	1.46	1.23–1.73

^1^ Adjusted for: Mother’s age, race/ethnicity, education, chronic diabetes and hypertension, prior uterine surgery, gravidity, gestational diabetes, pregnancy hypertension, placental problems, pregnancy-associated bleeding, father’s age, race and education, infant sex, and fertility group.

**Table 3:** Mediation analysis with Placental abnormalities as mediator.

Fertility Group	Total Effect				Direct Effect				Indirect Effect				%Mediation	
	LPTB		EPTB		LPTB		EPTB		LPTB		EPTB		LPTB	EPTB
	OR	95% CI	OR	95% CI	OR	95% CI	OR	95% CI	OR	95% CI	OR	95% CI		
Fertile	ref		ref		ref		ref		ref		ref			
ART														
All ART	1.47	1.37–1.58 ‡	1.72	1.52–1.93 ‡	1.36	1.26–1.46 ‡	1.45	1.28–1.62 ‡	1.08	1.07–1.09 ‡	1.19	1.15–1.22 ‡	23.27 ‡	37.27 ‡
Fresh	1.48	1.36–1.60 ‡	1.71	1.48–1.94 ‡	1.38	1.26–1.49 ‡	1.44	1.25–1.62 ‡	1.08	1.06–1.09 ‡	1.19	1.16–1.23 ‡	21.83 ‡	38.87 ‡
Frozen	1.41	1.21–1.62 ‡	1.61	1.24–1.98 ‡	1.31	1.12–1.49 ‡	1.39	1.08–1.70 ‡	1.08	1.06–1.10 ‡	1.16	1.10–1.21 ‡	25.36 ‡	35.91 ‡
Autologous egg	1.45	1.34–1.56 ‡	1.69	1.48–1.89 ‡	1.34	1.24–1.44 ‡	1.42	1.25–1.60 ‡	1.08	1.07–1.09 ‡	1.19	1.15–1.22 ‡	24.02 ‡	38.39 ‡
Donor egg	1.69	1.32–2.06 ‡	1.74	1.13–2.34 ‡	1.58	1.23–1.92 ‡	1.48	0.97–1.98 ‡	1.07	1.04–1.10 ‡	1.18	1.09–1.26 ‡	15.99 ‡	35.15 ‡
ICSI	1.48	1.32–1.65 ‡	1.61	1.31–1.91 ‡	1.39	1.23–1.55 ‡	1.34	1.09–1.59 ‡	1.07	1.05–1.08 ‡	1.20	1.15–1.25 ‡	19.45 ‡	44.40 ‡
No ICSI	1.46	1.30–1.62 ‡	1.74	1.44–2.04 ‡	1.35	1.21–1.49 ‡	1.46	1.21–1.71 ‡	1.08	1.06–1.10 ‡	1.19	1.15–1.24 ‡	23.83 ‡	37.75 ‡

LPTB = Late preterm birth; EPTB = Early preterm birth; OR = odds ratio; 95% CI = 95% confidence interval; ‡ = significant at *p* < 0.001.

**Table 4:** Mediation analysis with Pregnancy-Induced Hypertension as mediator.

Fertility Group	Total Effect				Direct Effect				Indirect Effect				%Mediation	
	LPTB		EPTB		LPTB		EPTB		LPTB		EPTB		LPTB	EPTB
	OR	95% CI	OR	95% CI	OR	95% CI	OR	95% CI	OR	95% CI	OR	95% CI		
Fertile	ref		ref		ref		ref		ref		ref			
ART														
All ART	1.40	1.30–1.50 ‡	1.52	1.34–1.70 ‡	1.36	1.26–1.46 ‡	1.45	1.28–1.62 ‡	1.03	1.02–1.04 ‡	1.04	1.03–1.06 ‡	9.36 ‡	12.43 ‡
Fresh	1.39	1.28–1.51 ‡	1.46	1.27–1.65 ‡	1.38	1.26–1.49 ‡	1.44	1.25–1.62 ‡	1.01	1.00–1.02 ‡	1.02	1.01–1.03 ‡	4.09†	5.65†
Frozen	1.42	1.21–1.62 ‡	1.58	1.22–1.94 ‡	1.31	1.12–1.49 ‡	1.39	1.08–1.70 ‡	1.08	1.06–1.10 ‡	1.14	1.11–1.17 ‡	25.94 ‡	32.64 ‡
Autologous egg	1.37	1.26–1.47 ‡	1.47	1.29–1.65 ‡	1.34	1.24–1.44 ‡	1.42	1.25–1.60 ‡	1.02	1.01–1.03 ‡	1.03	1.02–1.05 ‡	7.29 ‡	10.06 ‡
Donor egg	1.77	1.38–2.16 ‡	1.74	1.14–2.33 ‡	1.58	1.23–1.92 ‡	1.48	0.97–1.98 ‡	1.12	1.09–1.15 ‡	1.17	1.12–1.23 ‡	24.54 ‡	34.98 ‡
ICSI	1.42	1.26–1.58 ‡	1.39	1.13–1.64 ‡	1.39	1.23–1.55 ‡	1.34	1.09–1.59 ‡	1.02	1.01–1.03 ‡	1.04	1.02–1.05 ‡	7.32 ‡	12.18 ‡
No ICSI	1.36	1.21–1.50 ‡	1.47	1.22–1.72 ‡	1.35	1.21–1.49 ‡	1.46	1.21–1.71 ‡	1.01	1.00–1.01 ‡	1.01	0.99–1.02 ‡	1.88	1.95

LPTB = Late preterm birth; EPTB = Early preterm birth; OR = odds ratio; 95% CI = 95% confidence interval; ‡ = significant at *p* < 0.001.

## Data Availability

Data from this study may not be shared per MDPH protocols.

## Supplementary Materials

The following are available online at https://www.mdpi.com/article/10.3390/jcm10081681/s1, Table S1: ICD 9 and 10 codes for maternal conditions, pregnancy risks and delivery complications.
