# Supplementary material for: Influence of Placental Abnormalities and Pregnancy-Induced Hypertension in Prematurity Associated with Various Assisted Reproductive Technology Techniques
Source: J Clin Med. 2021 Apr 14;10(8):1681. doi: 10.3390/jcm10081681 (PMC8070757; doi:10.3390/jcm10081681)
Supplement: Supplementary file 1 [file jcm-10-01681-s001.pdf]

Journal of
Clinical Medicine

MDPI

Article

Supplementary Material: Influence of Placental Abnormalities and Pregnancy-Induced Hypertension in Prematurity Associated with Various Assisted Reproductive Technology Techniques

Judy E. Stern, Chia-ling Liu, Sunah S. Hwang, Dmitry Dukhovny, Leslie V. Farland, Hafsatou Diop, Charles C. Coddington and Howard Cabral

Table S1. ICD 9 and 10 codes for maternal conditions, pregnancy risks and delivery complications.

Categories	Conditions	ICD-9 Code	ICD-10 Code
Maternal Conditions			
	Chronic diabetes	648.0, 250	E08, E09, E10, E11, E13<br>O24.0, O24.1, O24.3, O24.8
	Chronic Hypertension	401, 402, 403, 404, 405<br>642.0, 642.1, 642.2, 642.7	I10, I11, I12, I13, I14, I15, I16<br>O10, O11
Pregnancy Risks			
Pregnancy Induced Hypertension (PIH)	Pregnancy Hypertension	642.3, 642.9	O13, O16
	Preeclampsia/Eclampsia	6424, 6425, 6426	O11, O14, O15
	Gestational Diabetes	6488	O24.4, O24.9
	Uterine Bleeding	6413, 6418, 6419	O46, O67
Delivery Complications			
Placental abnormalities	Abruptio placentae	641.2, 762.1	O45, P02.1
	Placenta previa	641.0, 641.1	O44
	Vasa Previa	663.5	O69.4
	Placenta accreta	667.0	O43.21
	Rupture membrane premature (< 12 hours)	658.1, 761.1	P01.1
	Rupture membrane prolonged (> 24 hours)	658.2, 658.3	O42.1
